# Enhanced Antioxidant Activity of Bioactives in Colored Grains by Nano-Carriers in Human Lens Epithelial Cells

**DOI:** 10.3390/molecules23061327

**Published:** 2018-05-31

**Authors:** Yoon-Mi Lee, Young Yoon, Haelim Yoon, Sooji Song, Hyun-Min Park, Yu Young Lee, Hyunho Shin, Sung Won Hwang, Kyung-Jin Yeum

**Affiliations:** 1 Department of Integrated Biosciences, College of Biomedical and Health Sciences, Konkuk University, Chungju-si 27478, Korea; yoonmilee@kku.ac.kr (Y.-M.L.); lab_yyoung0418@naver.com (Y.Y.); limtiny@naver.com (H.Y.); ssj4037@naver.com (S.S.); loveangela0312@gmail.com (H.-M.P.); 2 Nanotechnology Research Center, Konkuk University, Chungju-si 27478, Korea; 3 Department of Central Area, National Institute of Crop Science, Rural Development Administration, Suwon 16429, Korea; leeyy260@korea.kr; 4 Department of Nano Science & Mechatronics Engineering, College of Science and Technology, Konkuk University, Chungju-si 27478, Korea; modydog1@naver.com; 5 Institute of Biomedical and Health Science, Konkuk University, Chungju-si 27478, Korea

**Keywords:** bioavailability, oxidative DNA damage, γH2AX, black rice with giant embryo, reduced graphene oxide

## Abstract

The use of phytochemicals for preventing chronic diseases associated with oxidative stress such as cataracts is hindered by their low bioavailability. The effects of nano-carriers on the antioxidant activities of extracts of black rice with giant embryo (BRGEx) and soybeans (SBx) have been determined in human lens epithelial B3 cells. Scanning (SEM) and transmission electron microscopy (TEM) demonstrated that rGO (reduced graphene oxide) has a flat surface unlike GO (graphene oxide), which has a distinctive wrinkled structure with defects. UPLC analysis revealed 41.9 μg/100 g of γ-oryzanols in water extract of BRGE, and 111.8 μg /100 g of lutein, 757.7 μg/100 g of γ-tocotrienol, 4071.4 μg/100 g of γ-tocopherol in 40% ethanol extract of soybeans, respectively. Even though a low concentration of BRGEx alone did not show any antioxidant activity in B3 cells, co-treatment of BRGEx with rGO together substantially reduced hydrogen peroxide and methylglyoxal-induced DNA damage, as determined by phosphorylated γH2AX. In addition, SBx with rGO also attenuated DNA damage. Furthermore, intracellular reactive oxygen species were significantly decreased by combining extracts of these colored grains with rGO. These results suggest a potential application of nanocarriers for enhancing the bioavailability of phytochemicals.

## 1. Introduction

Excessive production of reactive oxygen species (ROS) triggers oxidative stress, thereby impairing macromolecules such as lipids, DNA, carbohydrates and proteins. These disruptions increase the risk of various chronic diseases such as cancer, cardiovascular diseases, metabolic syndrome, and neuronal diseases. Thus, the byproducts of damaged macromolecules can serve as a biomarker for the risk of various chronic diseases [1,2,3,4]. When DNA double-strand break occurs, 8-hydroxydeoxyguanosine (8-OHdG) and phosphorylated γ-H2AX histone protein are upregulated. Several biomarkers such as malonialdehyde (MDA), oxidized low-density lipoprotein (oxLDL) and 4-hydroxy-2-nonenal (4-HNE) have been established to determine oxidation of lipids. In addition, protein carbonyls are well known to be elevated under oxidative stress conditions [5]. 

Previously it has been shown that the antioxidative and anti-inflammatory properties of grains play a preventative and therapeutic role against chronic diseases by attenuating inflammation [5,6,7]. The underlying link between grains and chronic diseases is mainly associated with bioactive components. In particular, colored grains have much higher amounts of phytochemicals such as anthocyanins and carotenoids than non-colored grains [8,9,10,11,12,13,14].

Black rice with giant embryo (BRGE) is a newly developed variety of black rice, which contains nutrients such as anthocyanins and γ-aminobutyric acid (GABA) [15]. In our previous study, we identified various fat-soluble bioactives such as α- and γ-tocophenols, α- and γ-tocotrienols, luteins, γ-oryzanol and -carotenes in the BRGE [16]. Previous reports have shown the biological effects of BRGE as an anti-obesity agent in leptin-deficient C57BL/6J-ob/ob mice [12], and antianxiety in mice [17].

In addition to colored rice, soybeans contain high amounts of phytochemicals such as isoflavones which have favorable effects on various chronic diseases such as obesity, diabetes, cardiovascular diseases, kidney diseases, and cancer [18,19,20]. Black soybeans additionally possess anthocyanins in the black peel [21,22], and have been studied for their preventive roles against brain dysfunction, autoimmune arthritis [23] and anti-obesity [24,25]. However, Socheongja, a widely consumed black soybean cultivar, has rarely been studied for its biological activities. 

Fat-soluble compounds are reported to be poorly absorbed in the body [26]. Therefore, the application of these bioactives as an intervention for chronic diseases associated with oxidative stress such as cataracts [27,28,29] is limited. It should be noted that reducing oxidative stress has been suggested as an intervention for preventing cataracts, one of the major eye diseases causing blindness in the elderly, instead of relying on expensive lens extirpation surgery [30].

Application of nanotechnology in food and nutritional sciences in addition to medicine is an emerging field [31]. Graphene and its derivatives such as graphene oxide (GO) and reduced graphene oxide (rGO) have been intensely studied due to the fact these new carbon nanomaterials possess desirable characteristics such as tunable physical-chemical properties, a two-dimensional planar structure, a large specific surface area, and biocompatibility with cells and animals [32,33,34]. It is not surprising that such nanomaterials have been utilized for drug delivery for improving their efficacy [33,35]. Here we utilize graphene derivatives as nanocarriers to increase the bioavailability of bioactives in extracts of BRGE and Socheongja soybeans, thereby promoting its antioxidant activity in human lens epithelial cells for the first time, to the best of our knowledge. 

## 2. Results and Discussion

### 2.1. Characterization of Graphene Oxide and Reduced Graphene Oxide

**Figure 1:**
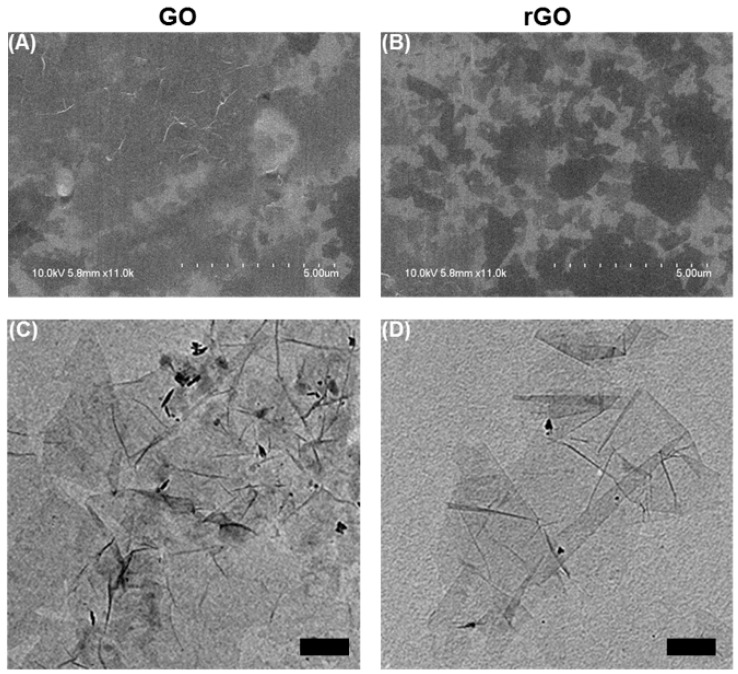
Top-view scanning electron microscopic (SEM) images of the (**A**) graphene oxide (GO) and (**B**) reduced graphene oxide (rGO), high resolution-transmission electron microscopic (HR-TEM) images of the (**C**) GO and (**D**) rGO.

The morphologies and characteristics of GO and rGO were determined by scanning electron microscopy (SEM) and high-resolution transmission electron microscopy (HR-TEM) as presented in Figure 1. SEM images of the 3D structure of GO (Figure 1A) and rGO (Figure 1B) are visible at high magnification and show their unique structures. The HR-TEM images (Figure 1C,D) present the detailed structural properties of GO and rGO with a porous 2D structure. GO has a distinctive wrinkled structure with many defects. On the other hand, the defects on rGO are clearly reduced from its thermal activation process. Even though the porous structure is still visible, rGO allows easy accessibility of bioactives.

### 2.2. Fat-Soluble Micronutrient Contents in Extracts of BRGE and Soybeans

We analyzed fat-soluble micronutrient contents in extracts of BRGE (BRGEx) and soybeans (SBx), respectively. We found 41.9 μg/100 g of γ-oyzanol in BRGEx. Then, we identified lutein (111.8 μg/100 g), γ-tocotrienol (757.7 μg/100 g) and γ-tocopherol (4071.4 μg/100 g) in SBx. These bioactive components are well known antioxidant components, thus leading to various biological functions for health benefits [36,37]. Further studies are necessary with regard to determination of functional groups or their purity in these extracts to understand the mechanism of actions for their antioxidant activities. 

### 2.3. Cytotoxicity Test of Extracts of Black Rice with Giant Embryo, Extracts of Soybeans, Graphene Oxide and Reduced Graphene Oxide

Black rice contains an extensive number of various phytochemicals that have been revealed in recent years. In particular, we have reported high contents of antioxidative phytochemicals in BRGE [16] and demonstrated its biological functions in attenuating metabolic disorders [12]. Considering oxidative stress as a key factor in cataract formation, antioxidant-rich BRGE may be able to prevent cataracts. We looked at the antioxidant activity of BRGEx in human lens epithelial B3 cells in order to determine the antioxidant capacity. However, the poor bioavailability of bioactive components such as anthocyanins and lutein in BRGE is a major hindrance in the utilization of dietary phytochemicals for preventing chronic diseases [38]. Sincegraphene-based nanomaterials have been emerging as drug delivery vehicles for the last several years [33,39], nano-carriers were introduced to enhance the delivery of phytochemicals into these cells. 

First, a water extract of BRGE, 40% ethanol extract of soybeans, GO and rGO were applied on human lens epithelial B3 cells to determine cytotoxicity, respectively. We found that BRGEx, SBx or rGO alone had no cytotoxicity to the human lens epithelial B3 cells at tested concentrations (0 to 100 µg/mL) (Supplementary Figure S1A,B,D). 

**Figure 2:**
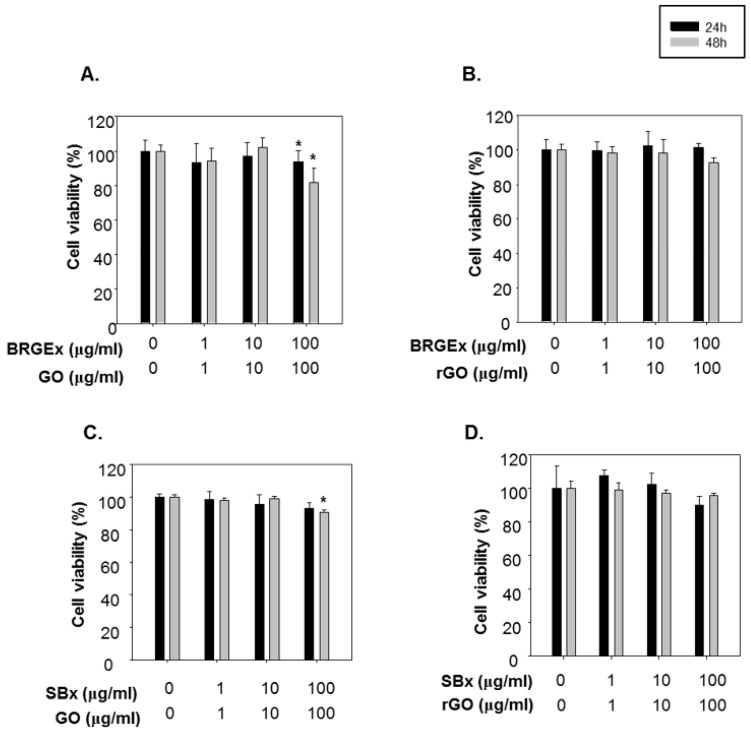
Effect of co-treatment of graphene oxide (GO) or reduced graphene oxide (rGO) with colored grain extract on cell viabilities in B3 cells. B3 cells were co-treated with (**A**) BRGE extract (BRGEx) and GO, (**B**) BRGE extract (BRGEx) and rGO, (**C**) soybean extract (SBx) and GO, (**D**) soybean extract (SBx) and rGO at the indicated dose for 24 h (black bar) or 48 h (gray bar), and then cell viabilities were determined using an MTT assay. * *p* < 0.05 vs. vehicle-treated cells.

However, GO had slightly decreased cell viabilities at 100 µg/mL after incubation for 48 h (Supplementary Figure S1C). When the cells were treated in combination of BRGEx with GO, cell viabilities were slightly decreased at 100 µg/mL (Figure 2A), whereas BRGEx and rGO co-treatment did not have any adverse effects while keeping dosing and incubation times the same (Figure 2B). Combined SBx and GO incubation for 48 h in human lens epithelial cells displayed cellular damage (Figure 2C), while there was no cytotoxicity in co-treatment of SBx and rGO (Figure 2D). Consequentially, we could find cytotoxicity only with the GO combination, and we hypothesize that a defective structure of GO can lead to reduced cell viabilities. Although the in vitro and in vivo safety of graphene derivatives has been highlighted previously [40,41], our data suggests that rGO was more reliable to use in human lens epithelial cells than GO. Based on our cell viability results, 10 µg/mL of GO and rGO were used to evaluate the contributory effect of nanomaterials on the antioxidant functions of BRGE and soybean extract.

### 2.4. Antioxidant Effects of Colored Grains (BRGEx and SBx) and Reduced Graphene Oxide

Oxidative stress by imbalanced redox status leads to damages in DNA, lipids, and proteins in the body. It is well known that such by-products in eye epithelial cells are closely related to the occurrence of cataracts [42]. We determined DNA damage by the phosphorylated ser139 residue on histone variant γH2AX in B3 cells.

**Figure 3:**
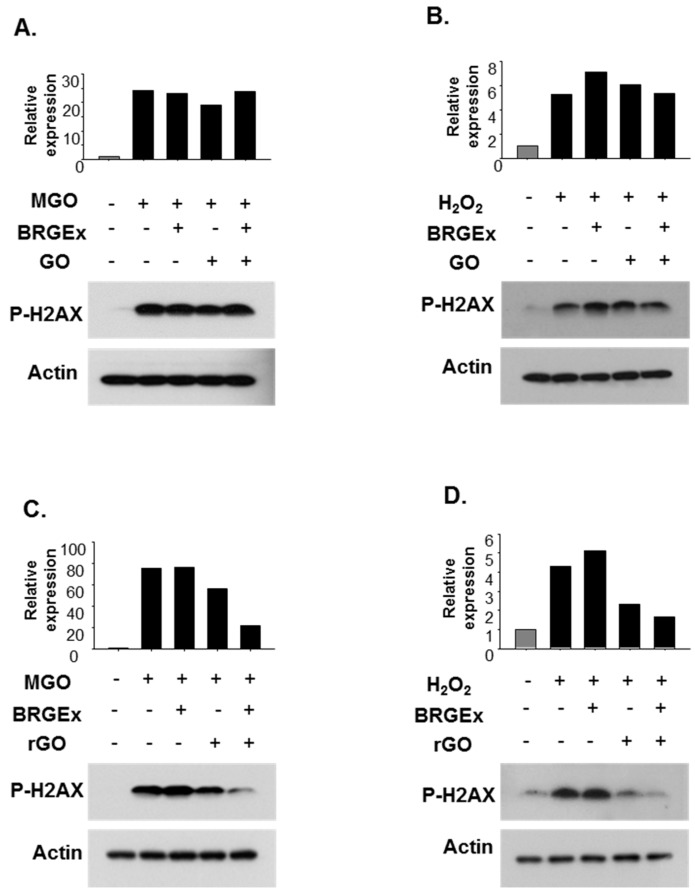
Effect of graphene oxide (GO) and reduced graphene oxide (rGO) with extract of black rice with giant embryo (BRGEx) on phosphorylated H2AX protein levels in B3 cells. B3 cells were pretreated with BRGEx (10 µg/mL) in the presence or absence of GO (10 µg/mL) (**A**,**B**) or rGO (10 µg/mL) (C,D) for 48 h, then 1 mM of MGO (**A**,**C**) and 100 µM of H_2_O_2_ (**B**,**D**) was added to the cells for 2 h. Phosphorylated h2AX protein levels were determined using Western blotting, and β-actin was used as loading control.

As shown in Figure 3, cells were pre-treated with BRGEx for 48 h, followed by treatment with methylglyoxal (MGO) as a reactive carbonyl species (RCS) and hydrogen peroxide (H_2_O_2_) as a representative reactive oxygen species (ROS) following previously used concentrations respectively [43,44]. As expected, phosphorylated H2AX protein levels were significantly up-regulated in cells stimulated with reactive carbonyl/oxygen species. However, when the B3 cells were pre-treated with BRGEx at a low concentration of 10 µg/mL before treatment with methylglyoxal or hydrogen peroxide, there was no significant difference in phospho-γH2AX protein levels for the B3 cells. Therefore, nano-carriers such as GO (Figure 3A,B) and rGO (Figure 3C,D) were introduced in an attempt to improve absorption of BRGEx into cells. There was no significant change with DNA damage when the cells were co-treated with BRGEx and GO in the presence of methylglyoxal (Figure 3A) or hydrogen peroxide (Figure 3B). Notably, concomitant treatment of BRGEx and rGO substantially reduced reactive carbonyl species- (Figure 3C) as well as reactive oxygen species- (Figure 3D) induced phosphorylated H2AX protein levels after 48 h of incubation. These results indicate that rGO has a supportive role for the biological efficacy of BRGEx.

**Figure 4:**
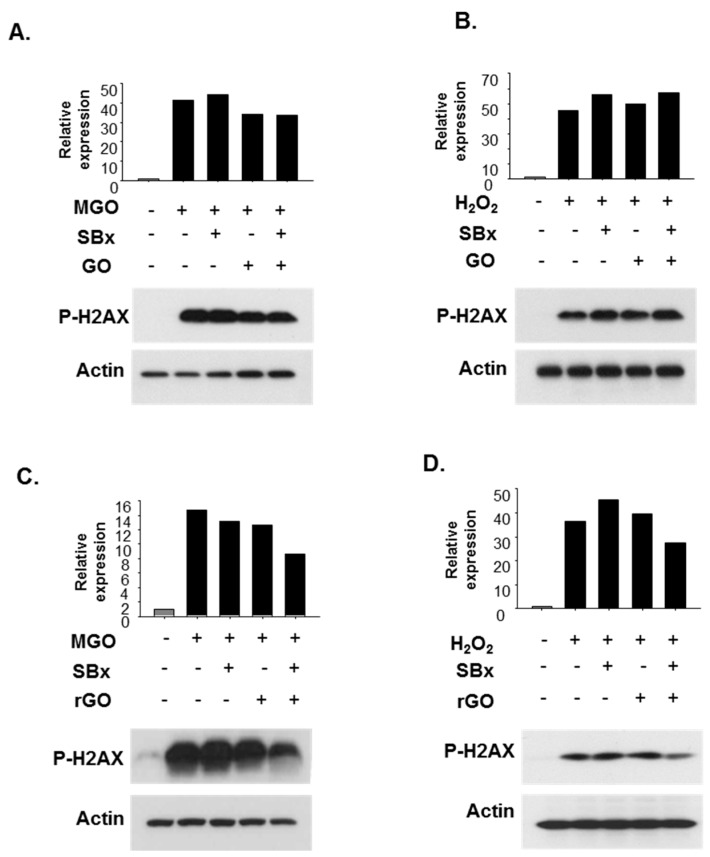
Effect of graphene oxide (GO) and reduced graphene oxide (rGO) with or without soybean extract (SBx) on phosphorylated H2AX protein levels in B3 cells. B3 cells were pretreated with SBx (10 µg/mL) in the presence of GO (10 µg/mL) (**A**,**B**) or rGO (10 µg/mL) (**C**,**D**) for 48 h then 1 mM of MGO (**A**,**C**) and 100 µM of H_2_O_2_ (**B**,**D**) was added to the cells for 2 h. Phosphorylated H2AX protein levels were determined using Western blotting, and β-actin was used as the loading control.

We next tried to confirm the increased bioefficacy of SBx using a graphene-based nano-carrier (Figure 4). Cells were pretreated with SBx at 10 μg/mL and nanocarriers such as GO or rGO. Co-treatment of rGO and SBx reduced DNA damage determined by phosphorylated γH2AX protein levels (Figure 4C,D) while GO did not have any effects on the SBx-treated cells (Figure 4A,B). These attempts demonstrated enhanced antioxidant activity of colored rice with rGO in human lens epithelial cells.

**Figure 5:**
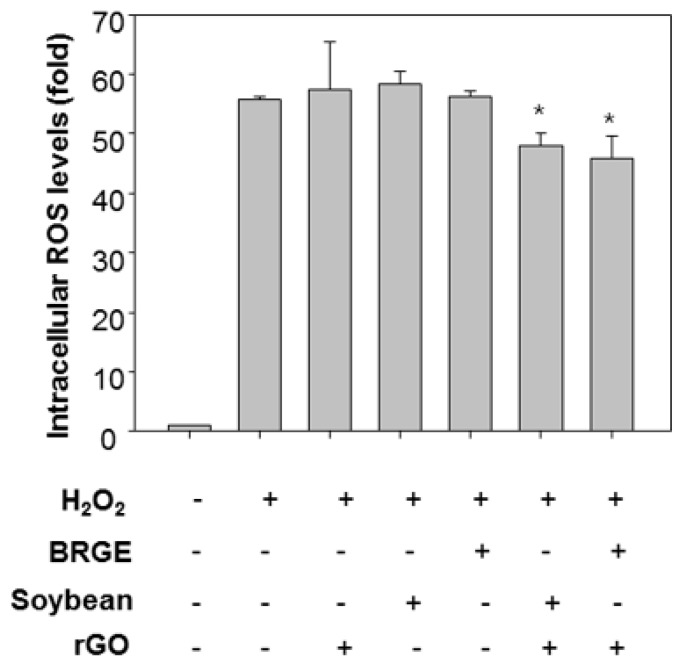
Effect of reduced graphene oxide with black rice with giant embryo/soybean extract on intracellular ROS levels. B3 cells were stained with DCF-DA (25 µM) for 30 min, and treated with each colored grain (10 µg/mL) in the presence or absence of rGO (10 µg/mL), and 100 µM of H_2_O_2_ 3 h. The fluorescence was measured at a wavelength of 483(ex)/535(em). * *p* < 0.05 vs. H_2_O_2_-treated cells.

Next, we assessed intracellular ROS levels to analyze antioxidant functions of BRGEx/SBx and rGO co-treatment. Cells were stained with DCF-DA which is fluorescent under oxidative condition [5]. Interestingly, co-treatment of BRGEx and rGO together reduced H_2_O_2_-induced intracellular ROS levels significantly as presented in Figure 5. Co-treatment of SBx and rGO also attenuated intracellular ROS levels in the cells. Thus, we confirmed rGO enhanced antioxidant activities of colored grains in human lens epithelial cells.

## 3. Materials and Methods

### 3.1. Materials

All chemicals and reagents were purchased from Sigma-Aldrich (St. Louis, MO, USA) unless otherwise noted. Water extract of BRGE was provided by the National Institute of Crop Science, Rural Development Administration, Republic of Korea. Two-hundred grams of BRGE was extracted with 200 mL of water for 5 days at room temperature. The extracts were then filtered and placed in a vacuum, followed by air-drying. The yield of the extract was 4.32%. Extract of Socheongja soybeans was provided by the National Institute of Crop Science, Rural Development Administration, Republic of Korea (Suwon, Republic of Korea). Grinded soybeans (40 g) were mixed with 40% ethanol solution (500 mL) for 24 h. The procedure was repeated three times, and the combined solution was filtered and dried at freezing conditions. The yield of extract of soybeans was 16.11%. Antibodies against phospho-γH2AX and β-actin were obtained from Cell Signaling Technologies (Danvers, MA, USA). 

### 3.2. Preparation and Structural Examination of Graphene Oxide and Reduced Graphene Oxide

GO was prepared from graphite powder using Hummers method [45,46,47,48] with minor modifications. To prepare the rGO, GO was deoxidized in a horizontal furnace at 200~350 °C for 2 h under Ar gas. About 0.05~0.5 g of GO powder was oxidized by ultrasonication in concentrated 10 mL H_2_SO_4_ and 30 mL HNO_3_ for 20 h, followed by dilution of the mixture with 250 mL of deionized water. The mixture was filtered using a 200 nm membrane to neutralize the acid. The size-reduced and purified 0.2 g GO powder was resuspended in 40 mL of deionized water, and the pH set at 8 using 1 M NaOH. The aliquot was transferred to a nitrogen-ambient furnace and heated at 250~300 °C for 10 h. To confirm the morphology and nanostructure, GO and rGO were characterized using scanning electron microscopy (SEM) and high resolution transmission electron microscopy (HR-TEM).

### 3.3. Ultra-Performance Liquid Chromatographic (UPLC) Analysis

Briefly, extracts of BRGE and soybeans extract were infused in chloroform: Methanol (2:1, *v*:*v*), respectively. After using the centrifuge, bottom layers were collected into a 12 × 75 mm tube. The BRGEx and SBx were further incubated in hexane, and centrifuged. At this time, upper layers were added to the first extracted solutions. These solutions were dried under nitrogen air, and the residue was resuspended with ethanol. Five microliters were loaded on UPLC (ACQUITY UPLC I-Class, Waters Co., Milford, MA, USA) equipped with a photodiode array detector, BEH C18 column (1.7 µm, 2.1 × 50 mm, Waters Co., Milford, MA, USA) and autosampler, and binary pump delivery system. The gradient method was set as the previously reported study and described in Table 1 [49]. The mobile phase A was acetonitrile/methanol (7:3, *v*/*v*), and the mobile phase B was water. γ-Tocopherol and γ-tocotrienol were detected at 292 nm, and lutein at 450 nm. All peaks were quantified under the curve of each standard, and established by retention time and its spectrum. The interassay coefficient of variation (CV) < 4% (*n* = 10), and the intraassay CV < 4% (*n* = 10) were confirmed.

### 3.4. Cell Culture

The human lens epithelial cell lines B3, immortalized by SV-40 viral transformation, were obtained from the American Type Culture Collection (Manassas, VA, USA), and grown in the minimum essential medium (Gibco, Waltham, MA, USA) supplemented with 20% fetal bovine serum (Gibco, Waltham, MA, USA), 100 U/mL penicillin, 100 µg/mL of streptomycin and 0.25 µg/mL amphotericin B. Cells were maintained in a humidified atmosphere with 5% CO_2_ at 37 °C. Cells were plated upon reaching 70 to 80% confluency when collecting samples. Cell were pretreated with BRGEx or SBx for 48 h, and then incubated in the medium containing MGO or hydrogen peroxide. Then, samples were collected for determination of DNA damage and intracellular ROS levels.

### 3.5. Western Blot Analysis

Cells were seeded into six well-plates, and incubated overnight. Then, cells were pretreated with extracts of BRGE or soybeans for 48 h, and further incubated in the presence of H_2_O_2_ or MGO following previously used concentrations for 2 h [43,44]. Cell lysates were collected using RIPA buffer (25 mM Tris-HCl, pH 7.6, 150 mM NaCl, 1% NP-40, 1% sodium deoxycholate and 0.1% SDS with protease inhibitors), followed by centrifugation for 15 min, 14,000 rpm under 4 °C. The supernatant was mixed with 4X sample buffer (250 mM Tris-HCl, pH 6.8, 8% SDS, 40% glycerol, 8% β-mercaptoethanol, and 0.02% bromophenolblue), and separated onto SDS-PAGE gels. Then, the gels were transferred to a PVDF membrane, blocked with 5% skim milk for 1 h at room temperature, and incubated with primary antibodies against phosphorylated H2AX and β-actin at 4 °C overnight. On the second day, the membrane was incubated with secondary antibodies conjugated with horse radish peroxidase (HRP). Afterwards, protein bands were determined by chemiluminescence (ECL). 

### 3.6. MTT Assay

Cells were seeded into 24 well-plates, and grown with the indicated treatment in Figure 2. After 24 h or 48 h, MTT solution (5 mg/mL) was added to the cells by 10% of medium volume (final concentration of MTT: 0.5 mg/mL), and incubated for 3 h in a humidified atmosphere with 5% CO_2_ at 37 °C. The medium was removed and solubilized with DMSO. The color of the end product was read at 540 nm (Molecular device, SPECTRAmax M2e, Sunnyvale, CA, USA). 

### 3.7. Measurement of Intracellular Reactive Oxygen Species (ROS) Levels

Cells were stained with 2′,7′-dichlorofluorescin diacetate (DCF-DA) for 30 min at 37 °C. Then, cells were treated with the experimental conditions described in figure legends. After 3 h, the fluorescence was read at wavelength of 485 (excitation)/535 (emission) by the fluorescence reader (Molecular device, SPECTRAmax M2e, Sunnyvale, CA, USA).

### 3.8. Statistics

Experiments were performed in triplicate. All data are presented as mean ± standard deviation (SD). Two-tailed unpaired student’s *t*-test and Mann-Whitney U test were used to analyze statistics between the two groups. When the *p*-value is below 0.05, data are considered as significantly different.

## 4. Conclusions

In this study, we demonstrated that rGO has a contributory effect for the extracts of BRGE and soybeans, exerting their antioxidant activities; whereas GO did not have any supportive role in human lens epithelial B3 cells. Both phosphorylated γH2AX protein levels and intracellular ROS levels decreased when both rGO and extracts of colored grans such as BRGE or soybeans were treated together. Therefore, utilization of nanocarriers can be a new strategy for enhancing bioavailability of phytochemicals. Further study is warranted for comparing its efficiency with conventional delivery systems such as liposomes, and determining its safety.

## Figures and Tables

**Table 1:** UPLC gradient method.

Time, min.	% A (ACN:MeOH = 7:3)	% B (Water)	Flow Rate (mL/min)
Initial	75	25	0.5
0.6	75	25	0.5
6.5	95.1	4.9	0.5
7.5	100	0	0.5
13.6	100	0	0.5
14.1	75	25	0.5
16.6	75	25	0.5

## Supplementary Materials

The following are available online.
