# Supplementary material for: Enhanced Antioxidant Activity of Bioactives in Colored Grains by Nano-Carriers in Human Lens Epithelial Cells
Source: Molecules. 2018 May 31;23(6):1327. doi: 10.3390/molecules23061327 (PMC6099946; doi:10.3390/molecules23061327)
Supplement: Supplementary file 1 [file molecules-23-01327-s001.zip › molecules-307688-SI.pptx]

## Slide 1
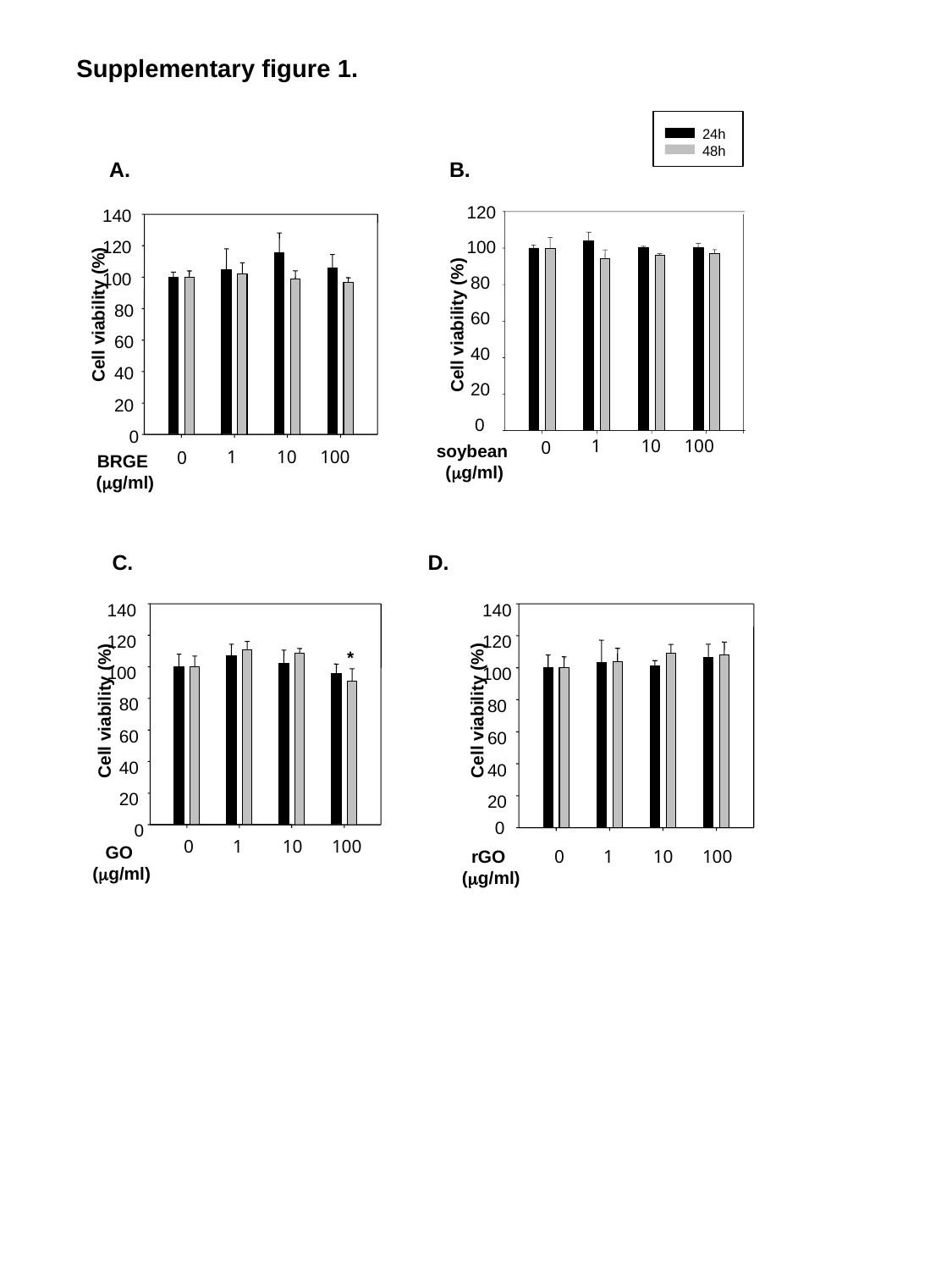

Supplementary figure 1.
24h
48h
B.
A.
120
140
120
100
80
60
40
20
0
100
80
Cell viability (%)
Cell viability (%)
60
40
20
0
1
10
100
0
soybean
(mg/ml)
1
10
100
0
BRGE
(mg/ml)
C.
D.
140
120
100
80
60
40
20
0
140
120
100
80
60
40
20
0
*
Cell viability (%)
Cell viability (%)
0
1
10
100
GO
 (mg/ml)
rGO
 (mg/ml)
0
1
10
100
